# Molecular investigation of tick-borne pathogens in dogs from Luanda, Angola

**DOI:** 10.1186/s13071-016-1536-z

**Published:** 2016-05-10

**Authors:** Luís Cardoso, Ana Cristina Oliveira, Sara Granada, Yaarit Nachum-Biala, Matan Gilad, Ana Patrícia Lopes, Sérgio Ramalho Sousa, Hugo Vilhena, Gad Baneth

**Affiliations:** Department of Veterinary Sciences, School of Agrarian and Veterinary Sciences, University of Trás-os-Montes e Alto Douro (UTAD), Vila Real, Portugal; Clínica Casa dos Animais, Luanda, Angola; Koret School of Veterinary Medicine, The Robert H. Smith Faculty of Agriculture, Food and Environment, The Hebrew University of Jerusalem, Rehovot, Israel; Animal and Veterinary Research Centre (CECAV), School of Agrarian and Veterinary Sciences, UTAD, Vila Real, Portugal; Department of Veterinary Medicine, Escola Universitária Vasco da Gama, Coimbra, Portugal; Hospital Veterinário do Baixo Vouga, Águeda, Portugal

**Keywords:** *Anaplasma platys*, Angola, *Babesia gibsoni*, *Babesia vogeli*, Canine vector-borne diseases, Dogs, *Ehrlichia canis*, *Hepatozoon canis*, Luanda, Polymerase chain reaction

## Abstract

**Background:**

No molecular data have been available on tick-borne pathogens that infect dogs from Angola. The occurrence of agents from the genera *Anaplasma*, *Babesia*, *Ehrlichia* and *Hepatozoon* was assessed in 103 domestic dogs from Luanda, by means of the polymerase chain reaction (PCR) and DNA sequence analysis.

**Results:**

Forty-six dogs (44.7 %) were positive for at least one pathogen. Twenty-one animals (20.4 %) were found infected with *Anaplasma platys*, 18 (17.5 %) with *Hepatozoon canis*, six (5.8 %) with *Ehrlichia canis*, six (5.8 %) with *Babesia vogeli*, one (1.0 %) with *Babesia gibsoni* and one (1.0 %) with an unnamed *Babesia* sp. The molecular frequency of single infections taken together was 37.9 % and that of co-infections with several combinations of two pathogens accounted for 6.8 % of the animals.

**Conclusions:**

This is the first report of *A. platys*, *B. vogeli*, *B. gibsoni*, *E. canis* and *H. canis* infections diagnosed by PCR in domestic dogs from Angola. The present study provides evidence that dogs in Luanda are widely exposed to, and at risk of becoming infected with, tick-borne pathogens. Further investigation is needed, including a larger number of animals, canine populations from other cities and provinces of the country, as well as potential vector ticks, aiming at better characterizing and controlling canine vector-borne diseases in Angola.

## Background

Angola is located in an area termed Middle Africa (United Nations geographic subregion). The country’s human population is slightly above 20 million, with a quarter living in the capital city of Luanda, which has a mild semi-arid climate, warm to hot and dry. The size of the canine population was estimated to be 480,000 at the country level in the year 2013, with a density of 0.39 dogs per square kilometer [1]. The number of dogs in Luanda has not been determined and they range from house-kept pets to free-roaming and stray animals.

Information on canine vector-borne disease (CVBD) agents at the local and regional levels allows veterinarians to better recognize the pathogens that can affect dogs, thus facilitating diagnosis and treatment [2, 3]. To date, no molecular data have been available on the prevalence or even the occurrence of tick-borne pathogens in dogs from Luanda, Angola. The hypothesis under testing in the current study was that owned dogs in Luanda are infected with a large number of different CVBD agents from the genera *Anaplasma*, *Babesia*, *Ehrlichia* and *Hepatozoon*.

## Methods

### Dogs and samples

One hundred and three pet dogs presented to a veterinary clinic in the city of Luanda, Angola, were sampled during January and February 2013. The age of dogs ranged from 3 to 168 months (median: 12 months; interquartile range: 7.3–48); and there were 61 males and 42 females. Owners provided their informed consent for inclusion of their animals in the study, which had been approved by the scientific council of Escola Universitária Vasco da Gama as complying with the Portuguese legislation for the protection of animals (Law no. 92/1995 and Decree-Law no. 113/2013).

Forty-nine apparently healthy dogs were presented for prophylactic procedures, including vaccination and deworming, or for elective surgery; 54 dogs clinically suspected of a CVBD had anorexia, weight loss, fever, dehydration, onychogryphosis, lymphadenomegaly, gastrointestinal alterations, jaundice, dermatological or ocular abnormalities, anemia, thrombocytopenia, leukocytosis or leukopenia, hyperproteinemia, and hyperglobulinemia. Sixty-two dogs had detectable ticks.

Blood was collected in EDTA and centrifuged, with two thirds of the plasma volume separated from cells, and the remaining plasma frozen together with cells at -20 °C. DNA was extracted from the concentrated blood samples using a commercial kit (E.Z.N.A.® Blood DNA Mini Kit, Omega Bio-Tek, Norcross, GA, USA), according to the manufacturer’s instructions.

### DNA amplification and sequencing

The detection of *Ehrlichia* and *Anaplasma* species was performed by screening all DNA samples first by a real time PCR assay targeting a 123 bp fragment of 16S rRNA gene (E.c 16S-fwd/E.c 16S-rev [4]). Positive samples were tested by a second conventional nested-PCR using the ECC and ECB primers targeting a 500 bp fragment of the 16S rRNA gene in the first round of PCR followed by a second round of PCR using *E. canis*-specific primers (Ecan/HE3 [5]) and *A. platys*-specific primers (ApysF/ApysR [5]) (Table 1). DNA extracted from an *E. canis* cell culture and DNA extracted from a dog infected with *A. platys* confirmed by PCR and sequencing were used as positive controls.

**Table 1:** Targeted organisms and list of primers used in this study

Target organism	Primer	Sequence	Fragment length (bp)	Reference
*Ehrlichia* spp.*/* *Anaplasma* spp.	E.c 16S-fwd E.c 16S-rev	TCGCTATTAGATGAGCCTACGT GAGTCTGGACCGTATCTCAGT	123	[4]
Anaplasmataceae	ECB ECC	CGTATTACCGCGGCTGCTGGCA AGAACGAACGCTGGCGGCAAGC	500	[5]
*Ehrlichia canis*	ECAN5 HE3	CAATTATTTATAGCCTCTGGCTATAGGA ATAGGGAAGATAATGACGGTACCTATA	400	[5]
*Anaplasma platys*	ApysF ApysR	GTCGAACGGATTTTTGTCGT TAGATCACCGCCTTGGTAGG	200	[5]
*Babesia* spp./ *Hepatozoon* spp.	Piroplasmid-F Piroplasmid-R	CCAGCAGCCGCGGTAATTC CTTTCGCAGTAGTTYGTCTTTAACAAATCT	400	[6]
*Babesia* spp.	Babesia18S-F Babesia18S-R	CCGTGCTAATTGTAGGGCTAATACA GCTTGAAACACTCTARTTTTCTCAAAG	551	[7]
*Hepatozoon* spp.	Hepatozoon18S-F Hepatozoon18S-R	GGTAATTCTAGAGCTAATACATGAGC ACAATAAAGTAAAAAACAYTTCAAAG	574	[7]

*bp*, base pairs

Molecular detection of *Babesia* and *Hepatozoon* species was performed by screening all DNA samples by a conventional PCR assay targeting a 400 bp fragment of the 18S rRNA gene (Piroplasmid-F/Piroplasmid-R [6]). In order to identify cases of co-infection, positive samples were tested by additional PCRs using primers specifically designed for the detection of a fragment of the18S rRNA gene of *Babesia* spp. (Babesia18S-F/Babesia18S-R [7]) and *Hepatozoon* spp. (Hepatozoon18S-F/Hepatozoon18S-R [7]) (Table 1). DNA extracted from a dog infected with *H. canis* and from another dog infected with *B. vogeli* confirmed by PCR and sequencing were used as positive controls.

Conventional PCR was performed in a total volume of 25 μl using the PCR-ready High Specificity mix (Syntezza Bioscience, Jerusalem, Israel) with 500 nM of each primers and sterile DNase/RNase-free water (Sigma, St. Louis, MO, USA). Amplification was performed using a programmable conventional thermocycler (Biometra, Göttingen, Germany). Initial denaturation at 95 °C for 5 min, was followed by 35 cycles of denaturation at 95 °C for 30 s, annealing and extension at 65 °C for 30 s (for ECC/ECB), 62 °C for 30 s (for ApysF/ApysR), 64 °C for 30 s (for Piroplasmid-F/Piroplasmid-R), 58 °C for 30 s (for Babesia18S-F/Babesia18S-R), 50 °C for 30 s (for Hepatozoon18S-F/Hepatozoon18S-R) and 10 cycles of 62 °C for 30 s followed by 25 cycles of 60 °C for 30 s for the ECAN5/HE3 primers, and final extension at 72 °C for 30 s. After the last cycle, the extension step was continued for a further 5 min. PCR products were electrophoresed on 1.5 % agarose gels stained with ethidium bromide and evaluated under UV light for the size of amplified fragments by comparison to a 100 bp DNA molecular weight marker.

Real time PCR was performed in a total volume of 20 μl containing 5 μl DNA, 400 nM of each primer, 10 μl Maxima Hot Start PCR Master Mix (2×) (Thermo Scientific, Epsom, Surrey, UK), 50 μM of SYTO9 solution (Invitrogen, Carlsbad, CA, USA) and sterile DNase/RNase-free water (Sigma, St. Louis, MO, USA), using the StepOnePlus real-time PCR thermal cycler (Applied Biosystems, Foster City, CA, USA). Initial denaturation for 5 min at 95 °C was followed by 40 cycles of denaturation at 95 °C for 5 s, annealing and extension at 59 °C for 30 s, and final extension at 72 °C for 20 s. Amplicons were subsequently subjected to a melt step with the temperature raised to 95 °C for 10 s and then lowered to 60 °C for 1 min. The temperature was then raised to 95 °C at a rate of 0.3 °C per second. Amplification and melt profiles were analyzed using the StepOnePlus software v2.2.2 (Applied Biosystems, Foster City, CA, USA).

Negative uninfected dog DNA, and non-template DNA controls were used in each run for all pathogens.

Positive PCR products were sequenced using the BigDye Terminator v3.1 Cycle Sequencing Kit and an ABI PRISM 3100 Genetic Analyzer (Applied Biosystems, Foster City, CA, USA), at the Center for Genomic Technologies, Hebrew University of Jerusalem, Israel. DNA sequences were evaluated with the ChromasPro software version 2.1.1 (Technelysium Pty Ltd., South Brisbane, QLD, Australia) and compared for similarity with sequences available in GenBank®, using the BLAST program (http://www.ncbi.nlm.nih.gov/BLAST/). The species identity found was determined according to the closest BLAST match with an identity of 97–100 % [8–10] to an existing GenBank® accession (Table 2).

**Table 2:** Vector-borne pathogens from the 46 positive dogs and their similarity with sequences deposited in GenBank®

Primers	Closest GenBank® accession	Identity (no. of dogs)	Pathogen	Dog’s code	New GenBank accessions
ECB/ECC +	KP844663	97–99 % (5)	*Ehrlichia canis*	L-002	KX082900
ECAN5/HE3	KP662546	100 % (1)		L-009	KX082901
ECB/ECC + ApysF/ApysR	KT357373	100 % (19)	*Anaplasma platys*	L-001	KX082895
	KP903295	99 % (1)		L-015	KX082896
	KF576218	97 % (1)		L-043	KX082897
				L-075	KX082898
				L-103	KX082899
Piroplasmid-F/ Piroplasmid-R	KP864658	99 % (5)	*Babesia vogeli*	L-018	KX082915
				L-019	KX082916
				L-024	KX082917
	LC012801	97 % (1)	*Babesia gibsoni*	L-036	KX082918
	KJ956782	97 % (1)	*Babesia* sp.	L-084	KX082919
	KJ868816	99–100 % (10)	*Hepatozoon canis*	L-008	KX082910
	DQ439540	99–100 % (2)		L-021	KX082911
	KP216462	100 % (1)		L-032	KX082912
	KT587790	100 % (1)		L-041	KX082913
	KP216425	99 % (1)		L-105	KX082914
	KC138532	97 % (1)			
Babesia18S-F/ Babesia18S-R	KJ494656	99–100 % (4)	*B. vogeli*	L-018	KX082902
	KP410276	98 % (1)		L-019	KX082903
				L-024	KX082904
Hepatozoon18S-F/ Hepatozoon18S-R	KP715302	98–99 % (12)	*H. canis*	L-008	KX082905
	DQ439540	99 % (1)		L-021	KX082906
	KP182934	99 % (1)		L-032	KX082907
	KP715303	99 % (1)		L-041	KX082908
				L-105	KX082909

### Data analysis

Exact binomial 95 % confidence intervals (CI) were established for proportions. Analyses were done using the StatLib.

## Results and discussion

Out of the 103 dogs, 21 (20.4 %; CI: 13.1–29.5 %) were found infected with *A. platys*, 18 (17.5 %; CI: 10.7–26.2) with *H. canis*, six (5.8 %; CI: 2.2–12.2) with *E. canis*, six (5.8 %; CI: 2.2–12.2) with *B. vogeli*, one (1.0 %; CI: 0.0–5.3) with *B. gibsoni* and another one (1.0 %; CI: 0.0–5.3) with an unnamed *Babesia* sp. (Table 3). Forty-six dogs (44.7 %; CI: 34.9–54.8) were found infected with at least one of the detected pathogens; and seven dogs (6.8 %, CI: 2.8–13.5) were found co-infected with two of the pathogens (Table 3). Table 2 displays the identification of canine vector-borne pathogens according to the similarity of their amplified sequences with those available in GenBank®.

**Table 3:** Single and co-infections with vector-borne pathogens among 103 dogs from Luanda, Angola, as determined by PCR and DNA sequencing

Agent(s)	Positive dogs		
	*n*	%	CI
Single infections	39	37.9	28.5–48.0
*Anaplasma platys*	16	15.5	9.2–24.0
*Babesia gibsoni*	1	1.0	0.0–5.3
*Babesia vogeli*	3	2.9	0.6–8.3
*Babesia* sp.	1	1.0	0.0–5.3
*Ehrlichia canis*	5	4.9	1.6–11.0
*Hepatozoon canis*	13	12.6	6.9–20.6
Co-infections	7	6.8	2.8–13.5
*A. platys* + *B. vogeli*	1	1.0	0.0–5.3
*A. platys* + *H. canis*	4	3.9	1.1–9.6
*B. vogeli* + *E. canis*	1	1.0	0.0–5.3
*B. vogeli* + *H. canis*	1	1.0	0.0–5.3
Single + co-infections (≥ 1 agent)	46	44.7	34.9–54.8

*CI*, 95 % confidence interval

To the best of our knowledge, this is the first report of *A. platys*, *B. vogeli*, *B. gibsoni*, *E. canis* and *H. canis* in dogs from Angola. The results of this study provide evidence for the presence of up to five distinct tick-borne pathogens among the canine population from the city of Luanda, which had previously not been molecularly documented, with *A. platys* and *H. canis* being the most prevalent. At least one tick-borne agent was detected in around 45 % of the dogs examined and, although exposure can vary according to the different pathogens, pet dogs are at a moderate to high risk of being infected with vector-borne agents at the local level.

All the canine pathogens detected in the present study at the species level share *Rhipicephalus sanguineus* (*sensu lato*) [11] ticks as their exclusive, possible or presumed vector. The fact that *A. platys* and *H. canis* were more frequently found than *Babesia* spp. and *E. canis* in dogs from Luanda might be related to the hypothesis that the local tick vector populations more frequently harbour some specific agents than others [12]. On the other hand, infections with more virulent agents, such as *E. canis* and *Babesia* spp., are less likely to have high frequencies due to the fact that hosts more often succumb to disease or are treated against it, with pathogen circulation thus being decreased [13]. The high frequency of *A. platys* and *H. canis* should be brought to the attention of veterinarians and dog owners in order to decrease the burden of the diseases those agents can cause in dogs. Detection and identification of pathogen species, either in single or in co-infection, are necessary for the treatment and prevention of CVBDs [2].

Ticks have not been identified in the scope of the present study, but it is presumed that some or even all of them could be *R. sanguineus* (*s.l*.). Indeed, these are the most widespread ticks in the world, being most abundant in temperate, subtropical and tropical climate regions [11]. *Anaplasma platys*, *B. vogeli*, *B. gibsoni*, *Babesia* sp., *E. canis* and *H. canis* were found in dogs with clinical signs compatible with a CVBD and may have contributed to causing them. Still, *A. platys*, *B. vogeli*, *E. canis* and *H. canis* were also found in dogs not clinically suspect of a CVBD, thus revealing subclinical infections.

All the agents could be found in dogs that had not travelled outside of the Luanda province. This fact suggests that these infections were locally acquired and, together with the diseases they cause, are endemic in the area of Luanda. Rather than having recently emerged, some of these infections have locally existed, as suggested by microscopic observation of Giemsa-stained blood smears and rapid serological tests (unpublished observations provide names of those who made these observations), but this is their first detection and confirmation at the molecular level.

In the present study, one dog was found infected with *B. gibsoni*. This animal was a clinically suspect one-year old Pit Bull-type male dog, with short hair length and no detectable ticks, that had received ectoparasiticides, lived outdoors and had not travelled to outside of the Luanda province. In the USA [14–16] and Australia [17], *B. gibsoni* infection has been found mostly in Pit Bull Terrier dogs. Indeed, studies in these countries indicate that direct dog-to-dog transmission is highly likely through bites and might even be the main mode of transmission among fighting dog breeds [15, 17]. In the present study, there were six other Pit Bull-type dogs and four of them were found infected with at least one CVBD agent, i.e. one with *A. platys*, another one with *B. vogeli* and two with *H. canis*.

The samples tested in the present study were collected in a veterinary medical centre from client-owned dogs. This circumstance could have biased the inclusion of a greater number of animals clinically suspect of a CVBD (n = 54; 52.4 %) compared with a lower proportion they may represent in the general canine population of Luanda and Angola. The frequency of infection with each pathogen should be regarded as an average value, taking also into account that the sampled dogs were well-cared for and may have not represented the overall canine population both at the national and city levels. Due to these facts, the prevalence of tick-borne agents in the overall populations of dogs from Angola and from the Luanda province and city might be higher [18].

This preliminary and geographically localized sample may have also limited the detection of a wider variety of tick-borne and other vector-borne pathogens. For example, *B. rossi*, which was not detected in this study, is known to be endemic in South Africa [13], Sudan [19], Nigeria [20] and Uganda [21]. In addition, the agent of human monocytic ehrlichiosis, *Ehrlichia chaffeensis*, was previously detected in dogs from Uganda [21] and in ticks collected from dogs in Cameroon [22]; and the agent of human granulocytic ehrlichiosis, *Ehrlichia ewingii*, was detected in dogs from Cameroon [23]. The species *Babesia canis* (*sensu stricto*), which is prevalent in Europe, where it is vectored by tick *Dermacentor reticulatus*, was found in a dog from Nigeria [24]. In the present study, a dog found infected with *A. platys* and *H. canis* had also been found PCR-positive and seropositive for *Leishmania infantum* and clinically affected by leishmaniosis. The frequency of canine *Leishmania* infection in the studied population was apparently low (i.e. 1.0 % by PCR and 1.9 % by serological direct agglutination test) [25].

Prevention of CVBDs largely relies on ectoparasite control [26], with the regular or long-lasting application of effective anti-vector products on individual dogs remaining the best approach to control infestations and associated diseases [27]. Prevention of *H. canis* infection should, in addition, rely on avoidance of ingestion of ticks. Most tick-borne pathogens of dogs, such as *Anaplasma* spp., *Babesia* spp. and *Ehrlichia* spp., are transmittable through blood product transfusions and infection with those pathogens should be screened in canine blood donors on a regular basis [28].

## Conclusions

In conclusion, the present study provides evidence that dogs in Luanda are widely exposed to and at high risk of becoming infected with tick-borne pathogens. This is the first report of *A. platys*, *B. vogeli*, *B. gibsoni*, *E. canis* and *H. canis* molecular detection and characterization in domestic dogs from Angola. Veterinarians as well as pet owners will benefit from being aware of the confirmed existence of these CVBD agents, in order to better diagnose, treat and prevent infections and their related diseases in dogs. Further investigation, including a larger number of dogs, canine populations from other cities and provinces of Angola, as well as potential vector ticks, is needed to better characterize CVBDs in the country.

### Ethics approval

This study was approved by the scientific council of Escola Universitária Vasco da Gama as complying with the Portuguese legislation for the protection of animals (Law no. 92/1995 and Decree-Law no. 113/2013).

## Abbreviations

CI: 95 % confidence interval
CVBD: canine vector-borne disease
PCR: polymerase chain reaction

## References

[1] OIE: World Animal Health Information Database (WAHIS) Interface. http://www.oie.int/wahis_2/public/wahid.php/Countryinformation/Animalpopulation (2013). Accessed 10 Mar 2016.

[2] Baneth G, Bourdeau P, Bourdoiseau G, Bowman D, Breitschwerdt E, Capelli G (2012). Vector-borne diseases – constant challenge for practicing veterinarians: recommendations from the CVBD World Forum. Parasit Vectors.

[3] Lee GK, Ignace JA, Robertson ID, Irwin PJ (2015). Canine vector-borne infections in Mauritius. Parasit Vectors.

[4] Peleg O, Baneth G, Eyal O, Inbar J, Harrus S (2010). Multiplex real-time qPCR for the detection of *Ehrlichia canis* and *Babesia canis vogeli*. Vet Parasitol.

[5] Rufino CP, Moraes PH, Reis T, Campos R, Aguiar DC, McCulloch JA (2013). Detection of *Ehrlichia canis* and *Anaplasma platys* DNA using multiplex PCR. Vector Borne Zoonotic Dis.

[6] Tabar MD, Altet L, Francino O, Sánchez A, Ferrer L, Roura X (2008). Vector-borne infections in cats: molecular study in Barcelona area (Spain). Vet Parasitol.

[7] Almeida AP, Marcili A, Leite RC, Nieri-Bastos FA, Domingues LN, Martins JR (2012). *Coxiella* symbiont in the tick *Ornithodoros rostratus* (Acari: Argasidae). Ticks Tick Borne Dis.

[8] Karagenc TI, Pasa S, Kirli G, Hosgor M, Bilgic HB, Ozon YH (2006). A parasitological, molecular and serological survey of *Hepatozoon canis* infection in dogs around the Aegean coast of Turkey. Vet Parasitol.

[9] Allen KE, Li Y, Kaltenboeck B, Johnson EM, Reichard MV, Panciera RJ (2008). Diversity of *Hepatozoon* species in naturally infected dogs in the southern United States. Vet Parasitol.

[10] Baneth G, Sheiner A, Eyal O, Hahn S, Beaufils JP, Anug Y (2013). Redescription of *Hepatozoon felis* (Apicomplexa: Hepatozoidae) based on phylogenetic analysis, tissue and blood form morphology, and possible transplacental transmission. Parasit Vectors.

[11] Dantas-Torres F, Latrofa MS, Annoscia G, Giannelli A, Parisi A, Otranto D (2013). Morphological and genetic diversity of *Rhipicephalus sanguineus* sensu lato from the New and Old Worlds. Parasit Vectors.

[12] Latrofa MS, Dantas-Torres F, Giannelli A, Otranto D (2014). Molecular detection of tick-borne pathogens in *Rhipicephalus sanguineus* group ticks. Ticks Tick Borne Dis.

[13] Penzhorn BL (2011). Why is Southern African canine babesiosis so virulent? An evolutionary perspective. Parasit Vectors.

[14] Birkenheuer AJ, Correa MT, Levy MG, Breitschwerdt EB (2005). Geographic distribution of babesiosis among dogs in the United States and association with dog bites: 150 cases (2000–2003). J Am Vet Med Assoc.

[15] Yeagley TJ, Reichard MV, Hempstead JE, Allen KE, Parsons LM, White MA (2009). Detection of *Babesia gibsoni* and the canine small *Babesia* ‘Spanish isolate’ in blood samples obtained from dogs confiscated from dog fighting operations. J Am Vet Med Assoc.

[16] Birkenheuer AJ, Levy MG, Stebbins M, Poore M, Breitschwerdt E (2003). Serosurvey of anti*Babesia* antibodies in stray dogs and American pit bull terriers and American staffordshire terriers from North Carolina. J Am Anim Hosp Assoc.

[17] Jefferies R, Ryan UM, Jardine J, Broughton DK, Robertson ID, Irwin PJ (2007). Blood, Bull Terriers and Babesiosis: further evidence for direct transmission of *Babesia gibsoni* in dogs. Aust Vet J.

[18] Noden BH, Soni M (2015). Vector-borne diseases of small companion animals in Namibia: Literature review, knowledge gaps and opportunity for a One Health approach. J S Afr Vet Assoc.

[19] Oyamada M, Davoust B, Boni M, Dereure J, Bucheton B, Hammad A (2005). Detection of *Babesia canis rossi*, *B. canis vogeli*, and *Hepatozoon canis* in dogs in a village of eastern Sudan by using a screening PCR and sequencing methodologies. Clin Diagn Lab Immunol.

[20] Adamu M, Troskie M, Oshadu DO, Malatji DP, Penzhorn BL, Matjila PT (2014). Occurrence of tick-transmitted pathogens in dogs in Jos, Plateau State. Nigeria Parasit Vectors.

[21] Proboste T, Kalema-Zikusoka G, Altet L, Solano-Gallego L, de Fernández Mera IG, Chirife AD (2015). Infection and exposure to vector-borne pathogens in rural dogs and their ticks, Uganda. Parasit Vectors.

[22] Ndip LM, Ndip RN, Ndive VE, Awuh JA, Walker DH, McBride JW (2007). *Ehrlichia* species in *Rhipicephalus sanguineus* ticks in Cameroon. Vector Borne Zoonotic Dis.

[23] Ndip LM, Ndip RN, Esemu SN, Dickmu VL, Fokam EB, Walker DH (2005). Ehrlichial infection in Cameroonian canines by *Ehrlichia canis* and *Ehrlichia ewingii*. Vet Microbiol.

[24] Kamani J, Sannusi A, Dogo AG, Tanko JT, Egwu KO, Tafarki AE (2010). *Babesia canis* and *Babesia rossi* co-infection in an untraveled Nigerian dog. Vet Parasitol.

[25] Vilhena H, Granada S, Oliveira AC, Schallig HD, Nachum-Biala Y, Cardoso L (2014). Serological and molecular survey of *Leishmania* infection in dogs from Luanda. Angola Parasit Vectors.

[26] Coles TB, Dryden MW (2014). Insecticide/acaricide resistance in fleas and ticks infesting dogs and cats. Parasit Vectors.

[27] Cardoso L (2010). Dogs, arthropod-transmitted pathogens and zoonotic diseases. Trends Parasitol.

[28] Wardrop KJ, Birkenheuer A, Blais MC, Callan MB, Kohn B, Lappin MR (2016). Update on canine and feline blood donor screening for blood-borne pathogens. J Vet Intern Med.

